# Utility of Repetitive Nerve Stimulation in Myopathies

**DOI:** 10.15844/pedneurbriefs-34-4

**Published:** 2020-02-25

**Authors:** Abigail Schwaede, Amber N. Buehner, Vamshi K. Rao

**Affiliations:** 1 Division of Neurology, Ann & Robert H. Lurie Children’s Hospital of Chicago, Chicago, IL; 2 Department of Pediatrics, Northwestern University Feinberg School of Medicine, Chicago, IL

**Keywords:** Neuromuscular Junction, Myopathy, Repetitive Nerve Stimulation

## Abstract

Investigators from the Mayo Clinic, Rochester, MN, evaluated 157 patients with confirmed myopathy who had electrodiagnostic studies done between January 2007 and May 2017.

Investigators from the Mayo Clinic, Rochester, MN, evaluated 157 patients with confirmed myopathy who had electrodiagnostic studies done between January 2007 and May 2017. Diagnosis of myopathy was confirmed by muscle pathology or genetic studies in those 157 patients. The study examined the frequency and electrophysiologic characteristics of decrement in these patients.

Of the 157 patients with confirmed myopathy on muscle pathology, 4 patients (2.55%) showed a significant decrement (greater than 10% decrement at 2Hz RNS with a train of 4 stimuli). These 4 patients, with age of onset of symptoms between 18 and 75 years of age, were ultimately diagnosed with the following: centronuclear myopathy (Patient 1), distal myopathy with nonspecific pathological findings (Patient 2), anti-synthetase (Jo-1) antibody-associated inflammatory myopathy as well as hydroxychloroquine associated myopathy (Patient 3), and hydroxycholorquine associated myopathy with minimal perimysial inflammatory reaction (Patient 4).

Patient 1 had improvement in muscle strength with a trial of pyridostigmine and no improvement with immunotherapy. Patient 2 and Patient 3 showed no response to pyridostigmine and were not treated with immunomodulatory therapy. Patient 3 showed no improvement with pyridostigmine. Patient 4 was not treated with any medications. [1]

COMMENTARY. In the evaluation of a suspected disease of the neuromuscular junction, repetitive nerve stimulation (RNS) is a time-honored technique to help confirm a defect in neuromuscular junction (NMJ) transmission. Decremental response can occur in a subset of patients with either hereditary or acquired myopathies. Additionally, multiple studies in adults and children have shown neuromuscular transmission defects and abnormal RNS in both animal models and human subjects that did not have a primary NMJ pathology. Aside from primary disorders of the NMJ, decrement can sometimes be seen in pediatric patients with motor neuron disease, muscular dystrophies, and myotonic disorders [2]. The mechanism of neuromuscular transmission defect is thought to be multifactorial and hypotheses include increased refractoriness associated with repetitive discharges, as seen in myotonic disorders, and a co-occurring primary defect at the junction, as seen in some hereditary muscle diseases.

Clinically differentiating congenital myasthenic syndromes, congenital myopathies and other disorders of the neuromuscular axis can be a diagnostic challenge for even an experienced pediatric neuromuscular clinician. Infants with myasthenic syndrome do not always present with fatigable weakness. Furthermore, those presenting with a defect in neuromuscular transmission can have a broader differential beyond a primary defect of the NMJ.

Repetitive nerve stimulation was not a technique commonly used in the work-up and evaluation of congenital myopathy but would be beneficial in children for identifying a defect in neuromuscular transmission. Low Frequency stimulation (2-5Hz) can aid in identifying post synaptic disorders whereas high frequency settings (20-50 Hz) can help with presynaptic disorders. An alternative technique to consider would be single-fiber EMG. Children found to have a decrement on RNS might also benefit from further analysis, such as presence of myasthenia gravis antibodies and genetic testing for congenital myasthenic syndromes. Even in the neonatal period, RNS can be extremely useful to help guide the initial work-up and reach a diagnosis in a timely manner. Furthermore, therapeutic strategies could be employed such as a trial of an AChEI along with cautious use of immunotherapy. Other therapies that could be considered include 3,4-DAP (amifampridine) and albuterol, as these have shown to be effective in some myasthenic syndromes and help enhance NMJ transmission.

Further studies in children with congenital myopathies will aid clinicians to better understand the benefits of RNS in myopathies.

## Disclosures

The authors have declared that no competing interests exist.
